# How to develop a comprehensive Mangrove Quality Index?

**DOI:** 10.1016/j.mex.2019.06.014

**Published:** 2019-06-20

**Authors:** Faridah-Hanum Ibrahim, Fatimah Mohd Yusoff, Anwar Fitrianto, Ahmad Ainuddin Nuruddin, Seca Gandaseca, Zaiton Samdin, Norizah Kamarudin, Siti Nurhidayu, Mohamad Roslan Kassim, Khalid Rehman Hakeem, Shamsuddin Ibrahim, Ismail Adnan, Awang Noor Abdul Ghani, Rhyma Purnamasayangsukasih Parman, Siti Balqis Abd Razak, Siti Aminah Ibrahim, Fareha Hilaluddin, Fatin Ramli, Nik Harun Al-Rashid Nik Zaidin

**Affiliations:** a Faculty of Forestry, Universiti Putra Malaysia, 43400, UPM Serdang, Selangor, Malaysia; b Faculty of Agriculture, Universiti Putra Malaysia, 43400, UPM Serdang, Selangor, Malaysia; c Department of Statistics, Faculty of Mathematics and Natural Science, Bogor Agricultural University, Indonesia; d Laboratory of Marine Biotechnology, Institute of Bioscience, Universiti Putra Malaysia, 43400, UPM Serdang, Selangor, Malaysia; e Institute of Tropical Forestry and Forest Product (INTROP), Universiti Putra Malaysia, 43400, UPM Serdang, Selangor, Malaysia; f Department of Biological Sciences, Faculty of Science, King Abdul Aziz University, 21589, Jeddah, Saudi Arabia; g Faculty of Economics and Management, Universiti Putra Malaysia, 43400, UPM Serdang, Selangor, Malaysia

**Keywords:** How to develop a comprehensive mangrove quality index?, Mangrove health, PCA, Indicator, Formulation

## Abstract

Currently, the available indices to measure mangrove health are not comprehensive. An integrative ecological-socio economic index could give a better picture of the mangrove ecosystem health. This method explored all key biological, hydrological, ecological and socio-economic variables to form a comprehensive mangrove quality index. A total of 10 out of 43 variables were selected based on principal component analysis (PCA). They are aboveground biomass, crab abundance, soil carbon, soil nitrogen, number of phytoplankton species, number of diatom species, dissolved oxygen, turbidity, education level and fishing time spent by fishers. Two types of indices were successfully developed to indicate the health status viz., (1) Mangrove quality index for a specific category *(MQIS_i_*) and, (2) Overall mangrove quality index (*MQI*) to reflect the overall health status of the ecosystem. The indices for the five different categories were mangrove biotic integrity index ($MQIS_{1}$), mangrove soil index ($MQIS_{2}$), marine-mangrove index ($MQIS_{3}$), mangrove-hydrology index ($MQIS_{4}$) and mangrove socio-economic index ($MQIS_{5}$). The quality of the mangroves was classified from 1 to 5 viz. 1 (worst), 2 (bad), 3 (moderate), 4 (good), 5 (excellent). These *MQI* class could reflect the quality of mangrove forest which could be managed with the objective of improving its quality. Advantages of this method include:

• PCA to select metrics from ecological-socioeconomic variables
• Formulation of *MQI* based on selected metrics
• Comprehensive index to classify mangrove ecosystem health

## Graphical abstract

**Figure:**
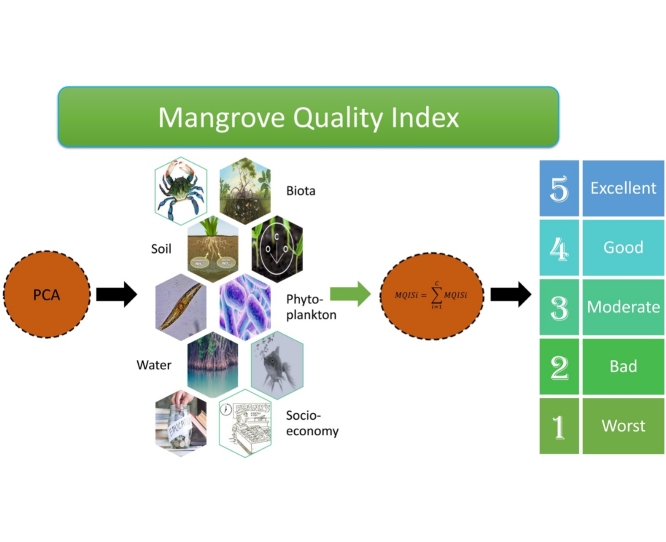

**Specifications Table**

**Table:** 

Subject Area:	*Agriculture and Biological Science*
More specific subject area:	*Forest management*
Method name:	*How to develop a comprehensive mangrove quality index?*
Name and reference of original method:	I. Faridah-Hanum, Fatimah M. Yusoff, A. Fitrianto, Nuruddin A. Ainuddin, Seca Gandaseca, S. Zaiton, K. Norizah, S. Nurhidayu, M.K. Roslan, Khalid R. Hakeem, I. Shamsuddin, Ismail Adnan, A.G. Awang Noor, A.R.S. Balqis, P.P. Rhyma, I. Siti Aminah, F. Hilaluddin, R. Fatin and N.Z.N. Harun. Development of a comprehensive Mangrove Quality Index (MQI) in Matang Mangrove: assessing mangrove ecosystem health (2018). *Ecological Indicators (Revised manuscript submitted)*
Resource availability:	*NA*

## Method details

### Rationale

Despite the various threats to mangrove ecosystems, systematic assessment of such changes has not been studied. There were no methods specifically for mangroves [1]. Current methods are not appropriate to be applied to mangroves because of the many unique characteristics confined to mangroves such as plants and animals, water and sediments. For instance, despite the fact that crabs play an important role in the mangrove ecology by affecting the chemical composition of soil as well as the growth and productivity of tree species, aerating the soil, removing harmful chemicals, and transporting nutrients, they were not included in current methods [2]. Due to complex interactions of factors in determining the health of a mangrove ecosystem, a comprehensive assessment of all integrating factors at the ecosystem level is needed to select appropriate indicators that could adequately reflect its real-time health status. However, not all factors can be included in establishing mangrove quality index. Appropriate strategies should be used in selecting effective indicator for the mangrove ecosystem health status. An integrative ecosystem-based approach should recognize not only the importance of interactions amongst many species, but also the roles of abiotic factors (environmental parameters) as well as social, economic and institutional perspectives [3]. Berezina et al. [4] used different physical, chemical and biotic variables such as water salinity, phosphorus, trace metals, polycyclic hydrocarbon, macroalgae biomass, phytoplankton and benthic organisms to make a comprehensive assessment of the environmental status of coastal habitats. As an example, Lopez and Fennessy [5] used the Floristic Quality Index for wetlands vegetation quality but its shortcoming was the exclusion of the abundance or dominance of plant species. Other measures of wetland quality include the estuarine rapid assessment procedure [1]. The index of biotic integrity [6] for wetlands was based on diversity and dominance but varied amongst vegetation classes. Integrating different types of data via satellite remote sensing, geographical information system (GIS) and modelling could be a useful approach to assess the status of a mangrove ecosystem [7]. Mangrove Vulnerability Index (MVI) using GIS was used to analyze social-ecological response to environmental changes and measure susceptibility to damage and capacity to cope or adapt [8]. Cao et al. [9] proposed the normalized difference vegetation index (NDVI) for a variety of remotely sensed imagery analysis related to vegetation. The NDVI was used to monitor shifting wetland vegetation [10,11] while [12] found NDVI useful to classify mangrove and non-mangrove areas.

Despite its importance, there still remains the issue of how to address the mangrove health. The development of Mangrove Quality Index (*MQI*) could be a way forward in determining mangrove health and provide solutions to rectify disturbances. In this way, effective mitigation measures can be administered rapidly to protect the resource sustainability. Besides, *MQI* can be a useful tool for managers to employ for decision making in matters pertaining to mangroves such as the intensity of rehabilitation, aquaculture project considerations and extent of resources’ protection. The *MQI* takes into account both biotic and abiotic variables including the socio-economy of the coastal community.

### Data acquisition

The methods involved in the development of the overall *MQI* are as follows: (1) classification of disturbance level using GIS and remote sensing; (2) Determination of socio-ecological mangrove ecosystem variables using PCA; (3) formulation of sub-index (*MQIS_i_*) and Overall *MQI*; (4) Summarizing the *MQI* and (5) Method validation

#### Classification of disturbance level using GIS and remote sensing

The study was conducted in Matang Mangrove Forest Reserve (MMFR) which is claimed as one of the best managed mangrove forests in the world. It is located in Peninsular Malaysia from 4^0^56’03.54″ N and 100^0^28’33.26″ E in the North and 4^0^32’10.81″ N and 100^0^37’40.54″ E in the South. With numerous rivers, large and small, MMFR is divided into four management zones viz., Kuala Sepetang North, Kuala Sepetang South, Kuala Trong and Sungai Kerang. MMFR has zones with logging activities for charcoal and poles but these deforested areas are then either replanted with suitable species or reforested naturally or both. Zones with lower intensity logging are used by the local community for their income by involving in commercial recreational activities. The different activities affect MMFR in terms of its biota, water quality, abundance of aquatic life and local inhabitants. The satellite image, Landsat 8, from USGS website [13] projected with Kertau Rectified Skew Orthomorphic (RSO) coordinate systems were used to classify the MMFR prior to data collection. Initial classification based on canopy density namely, dense, moderately dense, lowly dense and open area [14] was further supported by ground truthing of the different activities causing different levels of disturbance in MMFR. This led to the re-classification of MMFR into three categories of disturbance viz., least disturbed, moderately disturbed and highly disturbed areas which were used for this study.

#### Determination of socio-ecological mangrove ecosystem variables using PCA

A total of 43 variables comprising the biota, water, soil and socio-economy which were considered relevant to the project based on past studies and the National Water Quality Standards for Malaysia [15] were employed here (Table 1). These variables were subjected to principle component analysis (PCA) to determine the relevant metrics for the development of the *MQI*. In the process of determining the main metrics, no rotation strategy was used. It was based on correlation matrix. Two types of index were developed, which are *MQI* for each category (*MQIS_i_*) and the overall *MQI*.

**Table 1:** Socio-ecological variables used.

No.	Variables	Unit
**Biotic**		
1	Tree height	m
2	Basal area	m^2^/ha
3	Tree volume	m^3^/ha
4	Aboveground biomass	tonne/ha
5	Crab abundance	–

**Soil**		
6	Soil Carbon (C)	%
7	Soil Nitrogen (N)	%
8	Soil Phosphorus (P)	μg g^−1^
9	Soil Potassium (K)	μg g^−1^
10	Soil Calcium (Ca)	μg g^−1^
11	Soil Magnesium (Mg)	μg g^−1^
12	Soil pH	–
13	Soil Sulphur (S)	%

**Marine-mangrove**		
14	No. of phytoplankton species	Number
15	Phytoplankton abundance	cells ml^−1^
16	No. of diatom species	Number
17	Diatom abundance	cells ml^−1^
18	No. of dinoflagellates species	Number
19	Dinoflagellates abundance	cells ml^−1^
20	No. of copepods species	Number
21	Copepods abundance	Ind. m^−3^
22	No. of jellyfish species	Number
23	Jellyfish abundance	Ind. m^−3^

**Hydrology**		
24	Electrical Conductivity (EC)	μS/cm
25	Dissolved Oxygen	(mg/L)
26	pH	–
27	Turbidity	NTU (Nephelometric Turbidity Unit)
28	Total Dissolved Solid	mg/L
29	Temperature	^o^C
30	Total Suspended Solid	mg/L
31	Chemical Oxygen Demand (COD)	mg/L
32	Biochemical Oxygen Demand (BOD)	mg/L
33	Salinity	%
34	Ammonia (NH_3_)	mg/L
35	Phosphate (PO_4_)	mg/L
36	Nitrate (NO_3_)	mg/L
37	Fecal Coliform	mg/L

**Socio-economy**		
38	Fish landing	Weight of fish catches (kg/day)
39	Time spent	Number of hours fished (hour)
40	Fishing effort	Number of days fished (days/week)
41	Income	Monthly income (USD/person)
42	Age	Fishermen's age (year)
43	Education	Fishermen's education level (no of years)

**Fig. 1:**
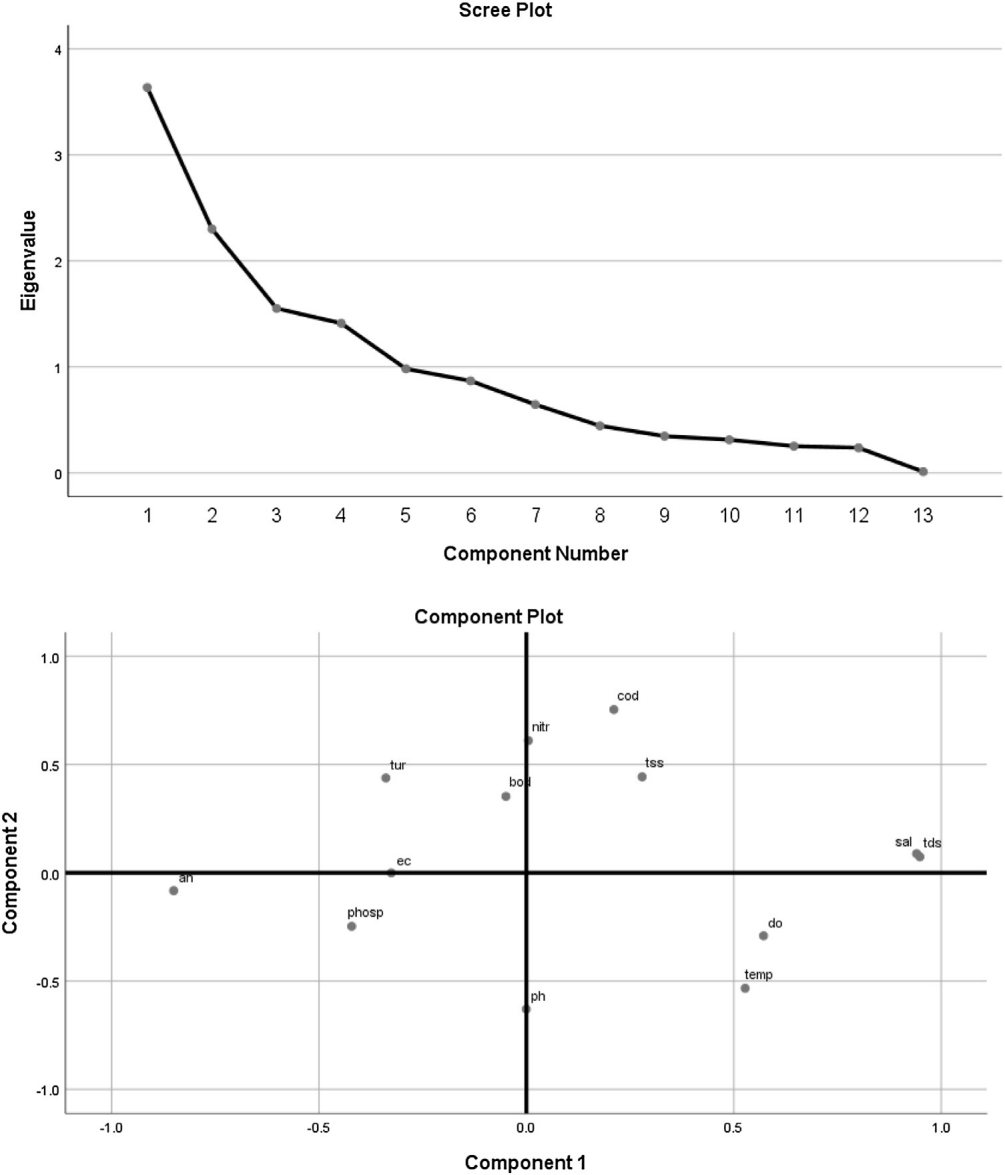
An example of PCA for hydrology variables in graphical form.

The PCA can also be shown in graphical form. Fig. 1 illustrates an example of PCA for hydrology variables.

The mangrove biotic integrity index was developed from the assessment of five biological variables which are tree height, basal area, tree volume, aboveground biomass and crab abundance. From the five variables subjected to PCA, the aboveground biomass of the mangrove trees and the abundance of crabs contributed to 91% of the cumulative variation, hence were chosen for the development of the overall *MQI*.

For the mangrove soil index, soil samples at four sites of different levels of disturbances were taken along 100 m length transect beginning from the riverbank and progressing inwards at 20 m interval plots and tested for Nitrogen (N), Carbon (C), Sulphur (S), Phosphorus (P), Potassium (K), Magnesium (Mg) and Calcium (Ca) in the laboratory using standard methods. Six variables of soil metrics were subjected to PCA analysis where soil nitrogen and soil carbon accounted for about 79% of the cumulative variation. These best two variables, soil nitrogen and carbon, were chosen for the development of the overall *MQI*.

The marine-mangrove health index was developed from the assessment of ten biological variables including the number of species and abundance of total phytoplankton, diatoms, dinoflagellates, copepods, and jellyfish. Monthly field samplings were carried out at the sampling stations. In the laboratory, all plankton samples were processed and prepared for the determination of species composition and abundance. All data were subjected to PCA analysis and the first two variables with the highest Eigen values (number phytoplankton and diatom species) were selected to contribute to the overall *MQI*.

To develop mangrove hydrology index, seven hydrological variables were identified i.e. electrical conductivity, dissolved oxygen, pH, turbidity, total dissolved solid, temperature and total suspended solid. Water samples were collected at three different locations (upstream, middle-stream and downstream). These seven variables were ordinated using PCA and only two most important variables were used to develop hydrological index i.e. turbidity and dissolved oxygen (DO).

Meanwhile for the mangrove socio-economic index, six variables were identified i.e. fish landing, time spent, fishing effort, income, age and education to develop socio-economic index. This study involved interviews of 300 local fishermen around MMFR at several jetties during December 2015. These six socio-economic variables were ordinated using PCA. Only two most important variables were used to contribute to the overall *MQI*, which were time spent and education level.

#### Formulation of sub-index (*MQIS_i_*) and overall *MQI*

The *MQIS* comprise of five socio-ecological categories, namely mangrove biotic integrity, mangrove soil, marine-mangrove, mangrove hydrology, and mangrove socio-economic indices. The steps to formulate these are as follows:

1 Conduct Principal Component Analysis (PCA) of all variables in a category,
2 Identify few most important variables in the category. The most important variables are characterized by the highest score component of each principal component (PC), The selected variables are also easy to measure and not highly technical.
3 Identify proportion of variability which can be explained by each important component. Let’s name the proportion as $p_{i}$,
4 Multiply the proportion in Step 3 by the corresponding score component, $Sc_{i}$, of each important metric and we have $pSc_{i}=p_{i}\times Sc_{i}$,
5 Calculate the summation of Step 4 for from all important metrics, $w_{T}=\sum pSc_{i}$,
6 Calculate weight, *w*_i_, of each important metric by dividing its corresponding $pSc_{i}$ by $w_{T}$, or $w_{i}=\frac{pSc_{i}}{w_{T}}$
7 Calculate mean, $\bar{x}$ and standard deviation, s of important variables (metrics) in Step 2.
8 *MQI* for a category is developed by standardizing a particular measurement. It is done to make sure that all important metrics have the same range of measurement although it originally comes from various units, i. $z_{i}=\frac{x_{ij}-{\bar{x}}_{i}}{s_{i}}$
9 Multiply the weight, wi (Step 6) with its corresponding $z_{i}$ and then multiplied by 2, since there are 2 important metrics need to be selected.
10 MQI Score *(MQIS)* of certain category is calculated using the following formula: a. $MQIS_{i}=\sum_{i=1}^{j}\;2\;w_{i}z_{i}$
11 *MQI* for *i*th category ($MQIS_{i}$) is shown as follows, where 1 (worst), 2 (bad), 3 (moderate), 4 (good), 5 (excellent).

MQISi= 1 if MQISi<-1.52 if-1.5 ≤MQISi ≤ -0.53 if-0.5 ≤MQISi ≤0.54 if 0.5 ≤MQISi ≤1.55 if MQISi>1.5

Where, i = 1, 2, …,5 represent the *i*th category as follows: $MQI_{1}$ (mangrove biotic integrity index), $MQI_{2}$ (mangrove soil index), $MQI_{3}$ (marine-mangrove index), $MQI_{4}$ (mangrove-hydrology index), and $MQI_{5}$ (mangrove-socioeconomic index).

In order to obtain the overall *MQI*, Step 1-Step 10 is repeated for all categories. Then the summation of *MQIS_i_* in Step 10 for each category is calculated to obtain overall *MQI* (Step 12 and Step 13)

12. Overall $MQI=\;\sum_{i=1}^{c}MQIS,$ where *c* is the number of categories
13. The range of overall *MQI* is defined as follows.

MQI= 1 if MQI<-1.52 if-1.5 ≤MQI ≤ -0.53 if-0.5 ≤MQI ≤0.54 if 0.5 ≤MQI ≤1.55 if MQI >1.5

where 1 (worst), 2 (bad), 3 (moderate), 4 (good), 5 (excellent)

#### Summarizing the *MQI*

The *MQIS* values for the three different level of disturbance in Matang Mangrove Forest Reserve viz., least disturbed, moderate disturbed and most disturbed is shown in Table 2. The first part of the table shows the environment component (*MQI*e) of the overall *MQI* while the second part consist of socio-economic component (*MQI*se).

**Table 2:** Summary of *MQI* for Matang Mangrove Forest Reserve.

No	Category	Variable		Environment *MQI* (*MQI*e) (Based on disturbance levels, a-c)		
				Least Disturbed (*MQI*ea)	Moderately Disturbed (*MQI*eb)	Most Disturbed (*MQI*ec)
1	Mangrove Biotic Integrity	Above Ground Biomass		4 (21.47; 39.00)^a^	3 (32.00; 70.00)	1 (24.00; 10.00)
		Crab Abundance				
2	Mangrove Soil	Soil N		5 (0.30; 9.52)	3 (0.40; 9.60)	1 (0.50; 15.00)
		Soil C				
3	Marine-Mangrove	No of Phytoplankton Species		5 (53.00; 38.00)	4 (27.00; 37.00)	2 (10.00; 12.00)
		No of Diatom Species				
4	Mangrove Hydrology	Dissolved Oxygen		4 (6.00; 7.00)	3 (4.00; 40.00)	2 (2.00; 91.00)
		Turbidity				
**Overall Environment *MQI* (*MQI*e)**				5	3	1
				**Socio-economic *MQI* (*MQI*se)**		
5	Mangrove Socio-Economic	Education (Years of Education)		4 (11.00, 10.00)		
		Time Spent (Hours)				
			**Overall *MQI***	**5**	**4**	**2**

a Random value for: (Variable 1; Variable 2).

#### Method validation

**Fig. 2:**
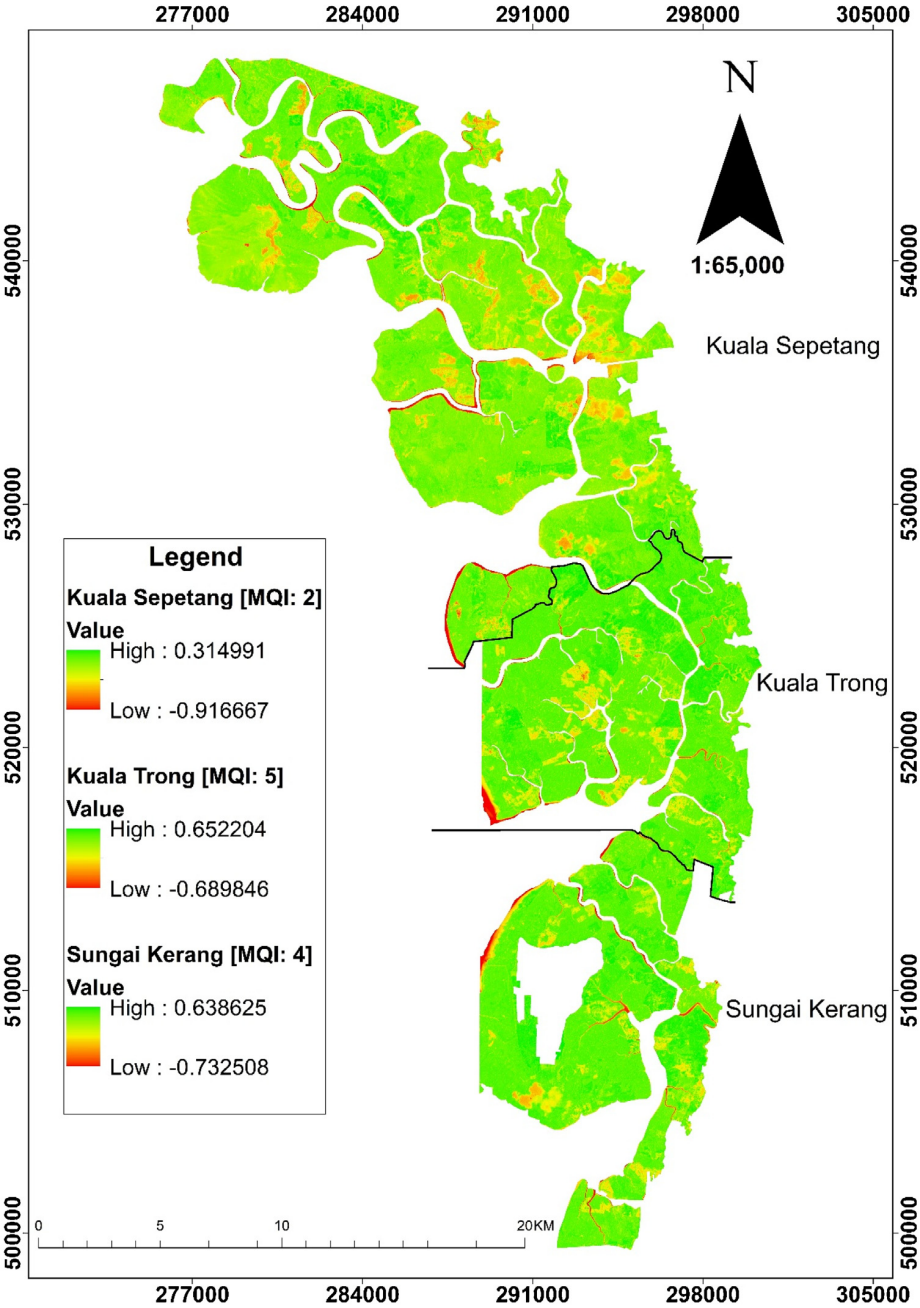
Computed NDVI image with three different level of disturbances of Matang Mangrove Forest.

The NDVI was used to find the vegetation index with band combinations of the remote sensing data by measuring the ability of vegetation to reflect (near-infra red-NIR channels) and absorb (red channels) electromagnetic radiation (EMR) with values from -1 to 1. If the vegetation has low reflectance (or low value) in the red channel and high reflectance in the NIR channel, this will yield a high NDVI value nearing 1, and vice versa. The NDVI was determined through SPOT image with 1.5 m resolution using ERDAS 2014 platform. In order to confirm the reliability of the developed *MQI*, the most recent image upon completion of sampling was obtained. The NDVI obtained was then used to confirm the degree of disturbance of the sampling sites. The ranking of these areas was validated; recent images analyzed through NDVI gave values of -0.916 to 0.315 for the area ranked as 2, -0.732 to 0.638 for the area ranked as 4, and -0.689 to 0.652 for area ranked as 5. The NDVI values nearing 1 in areas ranked as 4 and 5 showed the vegetation was denser while NDVI value nearing -1 in areas ranked as 2 showed the area being less dense with vegetation, hence supporting the overall *MQI* values obtained to indicate the Matang mangrove ecosystem health (Fig. 2).

## Conclusion

The method presented here shows how to develop the overall *MQI* which is comprehensive based on socio-ecological mangrove ecosystem variables. The advantage of this *MQI* is more integrated to show the mangrove ecosystem health. It can be used as an effective tool to manage mangrove ecosystem to ensure its quality and resilience are optimal for a sustainable ecosystem fulfilling the needs of local communities livelihood today and tomorrow. By increasing the *MQI* values, the quality of mangrove increases.
